# Marital status and mortality among Japanese men and women: the Japan Collaborative Cohort Study

**DOI:** 10.1186/1471-2458-7-73

**Published:** 2007-05-07

**Authors:** Ai Ikeda, Hiroyasu Iso, Hideaki Toyoshima, Yoshihisa Fujino, Tetsuya Mizoue, Takesumi Yoshimura, Yutaka Inaba, Akiko Tamakoshi, JACC Study Group

**Affiliations:** 1 Department of Public Health Medicine, Doctoral Program in Social and Environmental Medicine, Graduate School of Comprehensive Human Sciences, University of Tsukuba, 1-1-1 Tennoudai, Tsukuba-shi, Ibaraki, Japan; 2 Department of Social and Environmental Medicine, Graduate School of Medicine, Osaka University, 2-2 Yamadaoka, Suita-shi, Osaka, Japan; 3 Department of Public Health/Health Information Dynamics, Nagoya University Graduate School of Medicine, 65 Tsurumai-cho, Showa-ku, Nagoya, Japan; 4 Department of Clinical Epidemiology, Institute of Industrial Ecological Science, University of Occupational and Environmental Health, 1-1 Iseigaoka, Yahata-nishi-ku, Kitakyushu-shi, Fukuoka, Japan; 5 Department of Preventive Medicine, Graduate School of Medicine, Kyushu University, 3-1-1 Maidashi, Higashi-ku, Fukuoka-shi, Fukuoka, Japan; 6 Department of Epidemiology and Environmental Health, Juntendo University School of Medicine, 2-1-1 Hongo, Bunkyo-ku, Tokyo, Japan; 7 Department of Preventive Medicine/Biostatistics and Medical Decision Making, Nagoya University Graduate School of Medicine, 65 Tsurumai-cho, Showa-ku, Nagoya, Japan

## Abstract

**Background:**

Several studies have indicated a significant association between marital status and mortality risks. However, most of these studies have compared married and unmarried people without differentiating among single, divorced and widowed status. Moreover, gender differences in mortality rates associated with marital status have not been sufficiently clarified. With significant increases in the percentages of divorced and widowed people and a corresponding drop in the marriage rate in Japan during the past two or three decades, it can be expected that these changes will have a significant impact on mortality rates.

**Methods:**

This investigation used a prospective study of a total of 94,062 Japanese men and women aged 40–79 who completed self-administered questionnaires at baseline and during a followed-up of 9.9-years.

**Results:**

Compared with married men, never-married men showed higher risks of mortality from cardiovascular disease [relative risk (RR) = 3.05, 95% confidence interval (CI) 2.03–4.60], respiratory disease (RR = 2.43, 95%CI 1.27–4.63), external causes (RR = 2.18, 95%CI 1.05–4.54) and all causes (RR = 1.91, 95%CI 1.51–2.42) after adjustment for potentially confounding variables. For never-married women, there was a smaller but significantly higher risk of mortality from all causes (RR = 1.46, 95%CI 1.15–1.84). Divorced and widowed men showed moderately higher risks of mortality from cardiovascular disease, external causes and all causes compared with married men, but such a trend was not observed in women.

**Conclusion:**

Single status was associated with a higher risk of mortality than was married status for both men and women. Divorce and widowhood were associated with elevated risk for men, but not for women. These findings suggest single, divorce and widowhood status constitute potentially adverse health effects.

## Background

Marital status has been identified as an important social factor associated with mortality. Studies of this association conducted in the United States [1,2], European countries [3] and Japan [4,5] have shown that the risk of mortality for widowed, divorced, or single persons is 1.2 to 2.5-fold higher than for married persons. In the 1970s, an exceptionally high annual mortality rate was reported among Japanese singles (65 per 1000) compared to other countries (15–35 per 1000), possibly due to the high prevalence of tuberculosis [6,7]. One possible explanation for this finding is that, according to the customs associated with Japanese traditional arranged marriages, unhealthy individuals, such as those with a history of tuberculosis, could not get married, thus leading to a preponderance of healthy individuals in the married population [4].

In Japan, the divorce rate rose from about 2 percent between 1970 and 1974 to 10 percent between 1995 and 1999 with a corresponding drop in marriage rate from 92 to 69 percent [8], while the proportion of widowed persons rose from 6 to 21 percent [8]. A recent 7-year prospective study of 11,565 Japanese aged 40–69 found a risk of all-cause mortality among single men and women that was twice as high as that among their married counterparts, but the effects of divorce or widowhood on mortality were not be thoroughly examined in that study due to the limited number of deaths [9].

In general, married people are more likely to engage in positive and less likely to engage in negative health behaviors than widowed, divorced, or single people [10-13]. Some studies have suggested that the social ties, social networks, and/or social support, which marriage often provides, may reduce the risk of mortality [8,14,15]. Moreover, women in Japan tend to receive more social supports from the national government and from their relatives than do men. For instance, widows receive their deceased husband's pension as well as their own from the government, but widowers only their own pension. In addition, the national government provides child support for divorced women. Finally, many Japanese women return to live with their parents after a divorce (16%) or live with their children after their spouse's death (64%) [16]. Hence, there could be gender differences in adapting to the loss or the absence of a spouse among Japanese.

For the present study, we hypothesized that the risk of mortality from major diseases and all causes would be higher among widowed, divorced, and single persons than among those who are married. We also hypothesized that the excess mortality would be higher for men compared to women. The aim of this study was to analyze the association between marital status and mortality, and further to analyze if these associations were different between men and women.

## Methods

This study examined a total of 110,792 individuals (46,465 men and 64,327 women aged 40 to 79) who participated in the Japan Collaborative Cohort Study for Evaluation of Cancer Risk Sponsored by the Ministry of Education, Science, Sports and Culture (JACC Study). During 1988–1990, individuals living in 45 communities across Japan voluntarily participated in this study and completed self-administered questionnaires including lifestyles and medical histories of previous cardiovascular disease and cancer at baseline. Details of the study procedure were described previously [17]. Informed consent was obtained prior to completing the questionnaire. We used participant's responses to a question regarding marital status: "What is your marital status (married, widowed, divorced or single)?" However, a total of 1,486 men and 2,015 women were excluded from the analysis because the question on marital status was omitted in two communities (4%). A total of 39,471 men and 54,591 women (aged 40 to 79) provided a valid response to this question, and the response rate to this question was 88% for both men and women. Of this group, we also excluded 1,690 men and 2,308 women from the analysis due to a positive history of stroke, coronary heart disease or cancer at baseline. Therefore, the responses of 37,781 men and 52,283 women were included in the analysis of this study.

### Mortality surveillance

The subjects were followed from the date of the acceptance of the baseline survey through December 31, 1999. Since the information of the resident register is open to the general public under the resident registration law, investigators confirmed yearly residence status and survival with using residential registers, kept by a public health center in each of the study areas. Residency and death registration is required by Family Registration Law in Japan, and was believed to be complete across Japan. Death certificate diagnoses were provided by the Ministry of Health and Labor under permission from Welfare after Ministry of Internal Affairs and Communications granted permission. The underlying causes of deaths were defined according to the International Classification of Diseases, 9th Revision from 1988 to 1994, and 10th Revision from 1995 to 1999 for the National Vital Statistics. Therefore, all deaths that occurred in the cohort were confirmed by death certificates from a public health center, except for subjects who died after they moved from their original community, in which case the subject was treated as a censored case. The average follow-up period for the participants was 9.9 years. The Ethical Committees of the Nagoya University School of Medicine and the University of Tsukuba approved the present study.

### Statistical analysis

Statistical analyses were based on gender-stratified mortality during the follow-up period from 1989 to 1999. Each participant contributed person-time from the date of completed baseline questionnaire until the time of death or relocation, or December 31, 1999. Age-adjusted means and proportions of selected mortality risk factors were presented among the categories of marital status; the statistical testing for differences among marital status was conducted using analysis of covariance. Cox proportional hazards modeling was used to determine whether the marital status was significantly associated with stroke (ICD-9 codes 430 to 438, ICD-10 codes I60 to I69), coronary heart disease (410 to 414, I20 to I25), cardiovascular disease (390 to 459, I01 to I99), cancer (140 to 208, C00 to C97), respiratory disease (460 to 519, J00 to J99), external causes (800 to 999, S00 to T98) and all causes. We compared the mortality rates for people who were widowed, divorced, and single to those who were married (the reference group). The age-adjusted and multivariate-adjusted relative risks with 95% confidence intervals were calculated after adjustment for age and potential confounders. The confounding variables were obtained from the baseline questionnaire. Variables that were associated with both mortality and the categories of marital status were included in the multivariate analysis. The assumption of proportional hazards for all the selected confounders was tested by using both the time-dependent covariate method and the linear correlation test. We found no violation of the proportionality assumption. These confounding variables were age (in years), body mass index (gender-stratified quintiles), smoking status (never, ex-smoker, and current smokers of 1 to 19 and ≥20 cigarettes per day), alcohol intake (never, ex-drinker, and current drinkers of ethanol at 1 to 22, 23 to 45, 46 to 68, and ≥69 g per day), hours of walking (<1, 1 to 2, 3 to 4, and ≥5 hours per week), education (<13, 13 to 15, 16 to 18, ≥19 years), employment status (employed versus unemployed), and interest level in health screening (no, low, moderate, or high), having children (no or yes), history of hypertension (no versus yes) and history of diabetes (no versus yes). Psychological variables other than perceived mental stress were included as potential confounders in the additional analyses. These included hopelessness (definitely yes, yes, may be yes, or no), self-estimation of quick response (yes, may be yes, or no), sense of hurry (definitely yes, yes, may be yes, or no), anger (yes, may be yes, or no), joyfulness (definitely yes, yes, may be yes, or no), sense of being trusted (definitely yes, yes, may be yes, or no), and fulfillment (definitely yes, yes, may be yes, or no). Further stratification by age (40 to 64 years and ≥65 years) was also obtained in the analysis to assess effect modification. The presence of a statistical interaction was tested by using cross-product terms of marital status and stratifying variables. In addition, to reduce the potential effect of as-yet-undiagnosed diseases at baseline that could have affected marital status, we examined the marital status and mortality associations after exclusion of deaths during the first two years of follow-up.

## Results

We separately examined the four categories of marital status in relation to potential confounders for men and women (Table 1). Widowed men were approximately 10-year older, and divorced or single men were approximately 3 to 5 years younger than married men. Widowed, divorced, and single men were more likely to be unemployed, less educated, current smokers, and to have low interest in health screening than married men. The mean ethanol intake and the proportion of high-perceived stress were higher among widowed and divorced men and lower among single men compared with married men. Unmarried men were less likely to have a child than married men. The proportions of histories of hypertension and diabetes were higher among divorced men and lower among single men than married men. Compared with married men, unmarried men included a higher proportion of no quick response, no joyfulness, no fulfillment and not being trusted and lower proportion of being angry and in hurry, and hopelessness.

**Table 1:** Gender-stratified, age-adjusted mean values or prevalence of confounding factors atbaseline according to marital status

	Marital Status									
	
	Men					Women				
		
Marital Status	Married	Widowed	Divorced	Single	P-value	Married	Widowed	Divorced	Single	P-value
No. at risk	35,359	1,229	554	639		43,341	6,929	1,172	841	
Age, year	56.5	65.7	53.5	48.5	<0.001	55.5	65.6	56.2	55.9	<0.001
Body mass index, kg/m^2^	22.7	22.5	22.3	22.0	<0.001	22.9	22.9	22.7	22.1	<0.001
History of hypertension, %	19.8	19.4	20.3	16.3	0.20	21.4	23.9	19.9	17.7	<0.001
History of diabetes, %	6.5	6.3	10.5	5.5	0.004	3.6	4.3	4.4	4.2	0.06
Ethanol intake, g/day	34.2	35.7	34.6	30.7	0.01	9.9	11.1	20.6	13.9	<0.001
Walk 30 minutes or more/day, %	68.9	67.9	68.0	67.5	0.81	72.1	71.3	67.0	64.0	<0.001
Sport 5 hours or more/week, %	7.0	7.2	10.9	7.2	0.01	4.5	4.5	4.4	4.2	0.98
College or higher education, %	13.5	10.8	11.9	12.0	0.13	7.9	6.8	8.0	10.3	<0.001
Employed %	82.7	74.8	71.5	63.2	<0.001	38.1	42.2	47.1	45.7	<0.001
High Stress %	23.3	25.8	25.8	18.0	0.007	20.3	20.8	24.5	23.6	0.004
Current smoker, %	52.9	60.3	62.4	55.4	<0.001	4.4	7.7	21.5	9.9	<0.001
Low interest in health screening, %	18.0	21.6	23.7	27.8	<0.001	14.5	17.6	18.6	25.3	<0.001
No child, %	2.4	3.4	17.1	93.6	<0.001	2.8	3.9	9.7	96.3	<0.001
Psychological variables, %										
Hopelessness	6.7	14.3	14.7	21.5	<0.001	7.9	12.5	10.5	21.2	<0.001
No joyfulness	3.5	6.8	8.1	13.9	<0.001	4.1	5.4	8.3	9.1	<0.001
Not being trusted	10.0	15.1	16.1	23.1	<0.001	12.0	16.0	15.3	25.4	<0.001
No fulfillment	26.2	35.7	40.9	36.6	<0.001	31.2	43.7	51.7	45.8	<0.001
No quick response	8.1	10.5	9.0	21.2	<0.001	11.4	13.8	11.4	23.0	<0.001
Likely to be angry	89.1	83.7	90.6	81.6	<0.001	85.2	82.5	84.3	80.2	<0.001
Likely to be in hurry	92.5	88.6	91.2	80.9	<0.001	91.6	88.3	90.3	79.0	<0.001

Similar to men, widowed women were approximately 10 years older than married women, but there was no difference in mean age among divorced, single and married women. Unmarried women were more likely to be current smokers and to have low interest in health screening compared with married women. Unlike men, single women were likely to be more educated, employed, to have high perceived stress and to consume more ethanol compared with married women. The proportion having a history of hypertension was lower in divorced and single women and higher in widowed women, and that of diabetes was higher in unmarried women than married women. The results of psychological variables and the proportion of having a child in women were very similar to those in men.

During 892,998 person-years of follow-up, a total of 8,365 deaths (5,011 men, 3,354 women) occurred. Of these, 1,085 (585 men, 500 women) were attributable to stroke, 490 (294 men, 196 women) to coronary heart disease, 2,388 (1,326 men, 1,062 women) to cardiovascular disease, 3,339 (2,084 men, 1,255 women) to cancer, 857 (591 men, 266 women) to respiratory disease, and 364 (386 men, 248 women) to external causes.

Table 2 shows relative risks of mortality from cause-specific and all-cause mortality according to the marital status. Single men had approximately 2.0 to 3.5-fold higher risks of mortality from stroke, coronary heart disease and cardiovascular disease, respiratory disease, external causes, and all causes compared with married men. Widowed men had approximately 1.3 to 1.7-fold higher risks of mortality from stroke, coronary heart disease, cardiovascular disease, cancer, and external causes and all causes compared with married men. Divorced men had 1.6 to 2.5-fold higher risks of mortality from cardiovascular disease, respiratory disease, external causes and all causes compared with married men. For women, single had 1.7-fold higher risk of mortality from all causes and divorced had 2.3-fold higher risk of mortality from respiratory disease. Adjustment for potential confounding variables did not substantially alter these results.

**Table 2:** Gender-stratified relative risks (RR) and 95% confidence intervals of mortality due to stroke, coronary heart disease, cardiovascular disease, cancer, respiratory disease, external causes and all causes according to marital status.

	Men				Women			
		
Marital Status	Married	Widowed	Divorced	Single	Married	Widowed	Divorced	Single
**Stroke**	514	56	7	8	319	161	14	6
RR (95%CI) *	1.0	1.55(1.17–2.05)	1.18(0.56–2.49)	2.25(1.12–4.53)	1.0	1.13(0.93–1.39)	1.39(0.81–2.37)	0.91(0.40–2.03)
RR (95%CI) **	1.0	1.51(1.14–2.00)	1.13(0.53–2.39)	2.29(1.12–4.69)	1.0	1.10(0.90–1.35)	1.31(0.76–2.26)	0.87(0.38–1.97)
**Coronary Heart Disease**	254	27	6	7	120	69	4	3
RR (95%CI) *	1.0	1.70(1.14–2.55)	2.02(0.90–4.54)	3.49(1.64–7.42)	1.0	1.16(0.85–1.59)	1.03(0.38–2.80)	1.20(0.38–3.77)
RR (95%CI) **	1.0	1.60(1.07–2.40)	1.73(0.76–3.90)	3.46(1.57–7.58)	1.0	1.14(0.83–1.56)	0.82(0.29–2.26)	1.07(0.33–3.46)
**Cardiovascular Disease**	1158	121	22	25	670	349	22	21
RR (95%CI) *	1.0	1.53(1.26–1.85)	1.64(1.07–2.50)	2.98(2.01–4.44)	1.0	1.10(0.96–1.26)	1.02(0.67–1.57)	1.51(0.98–2.33)
RR (95%CI) **	1.0	1.46(1.20–1.77)	1.50(0.98–2.29)	2.95(1.96–4.45)	1.0	1.07(0.93–1.23)	0.95(0.62–1.46)	1.47(0.94–2.30)
**Cancer**	1903	139	25	17	912	287	30	26
RR (95%CI) *	1.0	1.27(1.07–1.51)	1.11(0.74–1.64)	1.02(0.63–1.65)	1.0	1.00(0.87–1.15)	1.13(0.78–1.62)	1.40(0.95–2.06)
RR (95%CI) **	1.0	1.22(1.02–1.45)	1.01(0.68–1.50)	0.91(0.56–1.47)	1.0	0.96(0.84–1.11)	1.02(0.70–1.47)	1.25(0.84–1.87)
**Respiratory disease**	519	49	13	10	158	89	12	7
RR (95%CI) *	1.0	1.13(0.84–1.52)	2.16(1.25–3.75)	3.32(1.78–6.22)	1.0	0.97(0.74–1.27)	2.27(1.26–4.08)	2.07(0.97–4.41)
RR (95%CI) **	1.0	1.05(0.78–1.41)	2.03(1.17–3.54)	2.36(1.24–4.51)	1.0	0.89(0.67–1.17)	2.12(1.17–3.85)	1.38(0.63–3.02)
**External causes**	342	25	11	8	184	55	2	7
RR (95%CI) *	1.0	1.64(1.08–2.47)	2.51(1.37–4.57)	2.01(0.99–4.07)	1.0	1.11(0.80–1.53)	0.38(0.10–1.54)	1.88(0.89–4.01)
RR (95%CI) **	1.0	1.53(1.01–2.31)	2.27(1.24–4.17)	2.18(1.05–4.52)	1.0	1.10(0.79–1.52)	0.38(0.09–1.53)	1.66(0.76–3.61)
**All causes**	4467	382	88	74	2257	943	77	77
RR (95%CI) *	1.0	1.37(1.23–1.53)	1.68(1.36–2.08)	2.08(1.65–2.62)	1.0	1.06(0.98–1.15)	1.11(0.89–1.40)	1.65(1.32–2.07)
RR (95%CI) **	1.0	1.30(1.17–1.45)	1.49(1.21–1.85)	1.85(1.46–2.34)	1.0	1.02(0.94–1.11)	1.01(0.80–1.27)	1.46(1.15–1.84)

*Adjusted for age.

** Adjusted for categories for multivariate adjustment were as follows: age, body mass index, smoking status, alcohol intake, education, minutes of walking, hours of doing sports, employment status, stress, having children, history of hypertension, and history of diabetes.

Furthermore, the excess risk of mortality due to cardiovascular disease and all causes associated with single status was more evident in men than in women (for interaction: p = 0.02 and p = 0.20, respectively). The excess risk of mortality from cardiovascular disease and all causes associated with widowhood was also more evident in men than in women (for interaction: p = 0.14 and p = 0.003, respectively). Furthermore, the excess risks of mortality from cardiovascular disease, external causes and all causes associated with divorced were more evident in men than in women (for interaction: p = 0.17, p = 0.02 and p = 0.02, respectively).

We repeated the analysis after excluding deaths during the first two years of follow-up to reduce the potential effect of as-yet-undiagnosed diseases on the associations, but we found no substantial changes in the above associations (not shown).

Relative risks of mortality from cardiovascular disease, cancer, and all causes were examined for men and women, further stratified by two age groups (40 to 64 and 65 to 79 years) (Table 3). The excess risk of mortality from all causes was more evident in younger single women (for interaction: p = 0.01) compared with older single women. The risk of mortality from all causes was higher in younger divorced or widowed men compared to older divorced or widowed men, but there were no statistical interaction between being divorced and age. The excess risk of cardiovascular mortality was more evident in younger single women (for interaction: p = 0.03) than older single women.

**Table 3:** Gender-stratified relative risks (RR) and 95% confidence intervals of mortality from cardiovascular disease, cancer, and all causes according to marital status.

	Men				Women			
		
Marital Status	Married	Widowed	Divorced	Single	Married	Widowed	Divorced	Single
Ages 40–64 y								
Person-years	277,605	5,030	4,497	5,751	358,675	29,012	8,764	6,949
**Cardiovascular Disease**	445	18	12	18	223	31	7	12
RR (95%CI)	1.0	1.46(0.91–2.35)	1.68(0.94–3.00)	3.15(1.90–5.22)	1.0	1.10(0.75–1.61)	0.93(0.43–2.02)	2.25(1.22–4.15)
**Cancer**	1,025	33	17	14	517	64	13	15
RR (95%CI)	1.0	1.16(0.82–1.64)	1.20(0.74–1.95)	1.12(0.65–1.93)	1.0	1.09(0.84–1.42)	0.93(0.53–1.62)	1.39(0.82–2.36)
**All Causes**	2,058	77	54	51	1,019	126	31	45
RR (95%CI)	1.0	1.40(1.11–1.76)	1.68(1.28–2.21)	1.82(1.36–2.43)	1.0	1.04(0.86–1.25)	1.06(0.73–1.52)	2.02(1.48–2.76)
Ages 65–79 y								
Person-years	70,287	5,787	682	362	77,063	38,189	2,751	1,595
**Cardiovascular Disease**	713	103	10	7	447	318	15	9
RR (95%CI)	1.0	1.43(1.16–1.77)	1.30(0.69–2.43)	2.14(1.00–4.56)	1.0	1.03(0.89–1.20)	0.91(0.54-1.53)	0.94(0.48–1.85)
**Cancer**	878	106	8	3	395	223	17	11
RR (95%CI)	1.0	1.26(1.03–1.55)	0.79(0.39–1.59)	0.62(0.20–1.95)	1.0	0.92(0.78–1.09)	1.10(0.67-1.80)	1.09(0.59–2.01)
**All Causes**	2,409	305	34	23	1,238	817	46	32
RR (95%CI)	1.0	1.27(1.12–1.43)	1.26(0.89–1.77)	1.84(1.21–2.79)	1.0	0.98(0.89–1.08)	0.99(0.73-1.33)	1.05(0.73–1.50)

Adjusted for categories for multivariate adjustment were as follows: age, body mass index, smoking status, alcohol intake, education, minutes of walking, hours of doing sports, employment status, stress, having children, history of hypertension, and history of diabetes.

## Discussion

In this large prospective analysis, it was found that, after adjustment of potentially confounding variables, the risk of mortality from cardiovascular disease, respiratory disease, external causes, and all causes was two- to three-fold higher for never-married than for married men. A smaller, but significantly higher mortality risk from all causes was observed for never-married women.

A higher risk of mortality from cardiovascular disease, external causes and all causes associated with divorce and widowhood was identified men, but not in women. The reason for this gender-based discrepancy may be that, unlike for men, the full-time employment rate was higher for both widowed and divorced women than for married women. Such labor force participation is likely to result in greater financial security and a more extensive social network. Compared with widowed or divorced men, moreover, widowed or divorced women receive more financial protection from the Japanese national government through widows' pensions and child support. Furthermore, Japanese women are generally unlikely to experience a drastic change in their social support network after becoming divorced or widowed [16]. These circumstances are likely to be responsible for the gender differences in mortality risks among Japanese.

The excess all-cause mortality risk associated with divorce or widowhood was larger in younger than in older men, a finding which is consistent with previously reported results [1,18,19]. In these previous studies, higher mortality was noted among widowed or divorced men and women soon after these life-changing events, but this excess risk declined with age. The excess mortality risks associated with being widowed or divorced and seen earlier in life gradually diminished later in life, probably because people in such groups tend to build certain health-enhancing environments that compensate for the loss or absence of a spouse.

An unusually high excess mortality rate was reported among Japanese singles due to the high prevalence of tuberculosis during the 1970s and 1980s [6,7]. There was no death from tuberculosis among the current study population, and there was only a small difference in the percentage of subjects with a history of tuberculosis between singles (16%) and married (11%). It can therefore be safely assumed that the excess mortality risks among singles in the present study were not attributable to tuberculosis infection, but rather to other factors such as psychosocial and environmental factors and/or lifestyles.

Marriage generally has a beneficial effect on health because it provides social support and social security and married persons are likely to experience less distress and to have a healthier lifestyle [12,13]. Marriage may also buffer against stress and thereby reduce the activation of neuroendocrine systems [20], which may lead to a reduction in the progression of atherosclerosis and other pathologic processes [21-24]. Previous prospective studies showed that weakened social ties and continued social isolation were associated with increased risk of mortality from coronary heart disease [14] and mortality from all causes [25] for both men and women.

In the study presented here, unmarried men and women tended to engage in more risk-promoting health behaviors (i.e., smoking, little physical activity, and low interest in health screening) and to experience more psychological distress (i.e., feelings of hopelessness and not being trusted, absence of joy and fulfillment) than did their married counterparts. In addition, unmarried men tended to have low socioeconomic status associated with low education and unemployment. On the other hand, more unmarried women were likely to be employed and to be more educated than their married counterparts.

Our study has several limitations. First, there is the possibility of a confounding effect from residual factors on the association between marital status and risk of mortality. Although adjustments were made for various risk factors for mortality, the possible influence of other risk factors and lifestyles cannot be ruled out. Second, differential follow-up among marital status categories may have affected the results since subjects who moved from their original community were treated as censored cases. In this study, the percentages of subjects who moved out of the communities examined were 3.0% for married, 4.0% for widowed, 9.6% for divorced and 8.1% for single men, and the corresponding percentages for women were 3.2%, 5.0%, 9.6% and 5.1%. Therefore, if it is assumed the mortality rates for censored subjects were similar or greater than those for uncensored subjects, the relationship of marital status and mortality may have been underestimated for both men and women. Third, since the study was not designed to analyze the effect of marital transitions, marital status measurements were not repeated during the follow-up. For example, misclassification resulting from the transition from marriage to widowhood may be more common among older than younger persons. Thus, the estimates for the association between marital status and mortality could be right-skewed for older subjects. Misclassification bias due to changes in marital status over time, especially from marriage to widowhood, may be particularly prominent among older persons [26].

The strengths of our study are its prospective design and large sample size, yielding high statistical power for detecting the effects of gender- and age-stratified marital status on mortality. It was also possible to clarify the associations between mortality and cardiovascular disease, cancer, respiratory disease, external causes as well as all causes. Finally, this study demonstrated gender differences in the excess risks of mortality.

## Conclusion

Single status was associated with a higher risk of mortality than was married status for both men and women. Divorce and widowhood were associated with elevated mortality risk for men, but not for women. These findings suggest single, divorce or widowhood status constitute potentially adverse health effects. The current decline in the marriage rate combined with increasing percentages of single, divorced, and widowed people may thus become an important public health issue, in terms not only of population aging but also of mortality.

## Abbreviations

RR = relative risk

95% CI = 95% confidence interval

ICD = International Classification of Diseases

## Competing interests

The authors declare that they have no competing interests.

## Authors' contributions

AI was involved in the analyses of the data and the manuscript writing. HI was involved in coordinating the study and advised about the concept of the article and data analysis, and the critical revision of the manuscript. HT, YF, TM, TY, YI, and AT were contributed in the critical revision of the manuscript. All authors read and approved the final manuscript.

## Pre-publication history

The pre-publication history for this paper can be accessed here:


